# SIRI-YOLO: A Foreign Object Detection Method for Belt Conveyors in High-Entropy Underground Scenes

**DOI:** 10.3390/e28060673

**Published:** 2026-06-11

**Authors:** Yi Liu, Yi Liu, Rengang Xue, Zixian Zhao, Jinping Xiao

**Affiliations:** 1 School of Artificial Intelligence, China University of Mining and Technology (Beijing), Beijing 100083, China; 13222719735@163.com (R.X.); sqt2310407045@student.cumtb.edu.cn (Z.Z.); 2 School of Mechanical and Electrical Engineering, China University of Mining and Technology (Beijing), Beijing 100083, China; zqt2300407092@student.cumtb.edu.cn; 3 Beijing Aerospace Changfeng ScienceTechnology Industry Group Co., Ltd., Beijing 100854, China; 13681581890@163.com

**Keywords:** belt conveyor, foreign object detection, multi-scale target, information entropy, boundary box regression

## Abstract

To address the poor detection performance in low-light underground coal mine belt conveyors caused by information entropy degradation and high background noise, as well as the difficulty in multi-scale target extraction due to uneven entropy distribution, this paper proposes an efficient foreign object detection model named SIRI-YOLO based on an improved YOLOv11n architecture. First, a Self-Calibrating Illumination Network (SCINet) is introduced to restore image information entropy and enhance low-light adaptability. Second, the C2PSA module is enhanced to C2PSA-IRMB by incorporating an Inverted Residual Mobile Block (IRMB), improving multi-scale feature utilization and reducing ineffective entropy increase. Third, an improved Reparameterized Generalized Feature Pyramid Network (RepGFPN) is adopted to strengthen the fusion of high-level semantics and low-level spatial features, reducing information entropy loss during feature pyramid transfer. Finally, the Inner-MPDIoU loss function is introduced to replace CIoU, achieving more accurate entropy minimization from a KL divergence perspective. Experimental results on a dataset containing large coal chunks and anchor rods show that SIRI-YOLO achieves 92.8% mAP@50, 59.4% mAP@50:95, 89.5% precision, and 87.2% recall, with only 2.92M parameters and 70.01 FPS, outperforming mainstream YOLO models. Furthermore, on the public ExDark low-light dataset, SIRI-YOLO improves mAP@50 by 4.2% over YOLOv11n, demonstrating strong generalization across different low-light and complex scenarios. The proposed method effectively handles uneven illumination, scale variation, and complex backgrounds, offering a practical solution for coal mine safety through system entropy reduction.

## 1. Introduction

The belt conveyor is a key piece of equipment in the field of coal transportation and is widely used in both underground and open-pit coal mining operations. Its continuous and efficient conveying capacity directly determines the productivity of coal mines and serves as critical infrastructure for ensuring industrial production continuity while reducing labor costs [1]. However, during the operation of belt conveyors, foreign objects such as anchor rods and large coal chunks are inevitably transported alongside the coal. The presence of such foreign materials can lead to various malfunctions, including belt deviation, slipping, idler failure, and belt tearing. These issues may result in equipment shutdowns and significant economic losses and, in severe cases, can even cause safety accidents such as fires and casualties [2]. Therefore, the timely and accurate detection of foreign objects on belt conveyors is essential to ensuring their safe and stable operation [3].

With the advancement of computer technology, traditional manual inspection can no longer meet the operational requirements of large belt conveyors for foreign object detection [4]. Currently, deep learning-based target detection has become the mainstream approach for this task. To address the challenges of low illumination and complex backgrounds in underground coal mines, researchers have proposed various improvement methods. A common strategy is to enhance the image before detection, using traditional techniques such as Retinex or histogram equalization, as well as deep learning-based generative adversarial networks (GANs) and curve estimation methods [5]. These methods have proven effective in improving contrast and reducing noise in dark images, but they typically require additional preprocessing steps. Another direction focuses on end-to-end detection network design. For example, Yin et al. [6] proposed PE-YOLO, which integrates Laplacian pyramid decomposition, detail processing, and low-frequency filtering modules into YOLOv3 to enhance different brightness components. Other studies introduce attention mechanisms and multi-scale features into the network architecture. Hashmi et al. [7] developed the FeatEnHancer module, which incorporates cross-layer attention into multi-scale convolutional features to enrich semantic representation. Meanwhile, Shi et al. [8] introduced a multi-scale convolution module with coordinate attention and a dynamic detection head based on YOLOv8, along with a non-monotonic focusing loss to improve adaptability to illumination changes. Some works also enhance low-light detection generalization through contrastive learning or domain adaptation. Lin et al. [9] proposed the SCDet framework, which uses supervised contrastive learning to mitigate the interference of low-light conditions, and Schutera et al. [10] adopted night-to-day image domain transfer to improve detection robustness. In summary, current methods can be roughly categorized into three types: (1) “image preprocessing + detection” approaches (Retinex, GAN, pyramid enhancement, etc.); (2) “attention or multi-task network structure improvement” approaches (multi-scale attention modules, dynamic heads, adaptive losses, etc.); and (3) “domain adaptation or adversarial” approaches (image transformation, contrastive learning, etc.). While these methods have achieved good results in various low-light and complex-background detection tasks, they often suffer from high computational cost or reliance on specific enhancement procedures. This paper integrates the above ideas and introduces innovations to the YOLO series to achieve more efficient target detection under low-light and complex background conditions.

The YOLOv11n model has improved structural design, enhanced feature extraction techniques, and optimized training methods, resulting in significantly improved detection accuracy and speed, thereby achieving a balance between detection accuracy and speed [11]. However, for the specific scenario of foreign object detection on underground belt conveyors, certain deficiencies remain, such as: (1) Effective information entropy degradation and an excessively high proportion of background noise entropy caused by lighting conditions, leading to image degradation and feature distortion: In underground coal mines, artificial light sources are dominant, leading to uneven illumination and low overall luminance. The captured images suffer from poor contrast and blurred details, causing the underlying texture features (as low-entropy signals) extracted by the model to be overwhelmed by high-entropy noise. This distortion leads to semantic drift during subsequent feature fusion and weakens the detection head’s response to foreign objects. (2) Insufficient multi-scale target perception due to uneven distribution of information entropy across different scales: Foreign objects include large coal chunks with significant scale variations (accounting for 10% to 30% of the image area) and slender anchor rods only a few pixels in width. A single-scale convolution kernel struggles to capture both the global structure of large targets and the local details of small targets, and the effective information of small targets is easily submerged by the high-entropy background. While simple attention mechanisms can model long-range dependencies, their high computational overhead makes them difficult to deploy on embedded devices. (3) Feature fusion misalignment in complex scenarios, which causes information entropy loss during feature pyramid transfer: The original FPN + PAN structure in YOLOv11n employs a relatively fixed interaction path between high-level semantics and low-level spatial details during layer-by-layer fusion. This makes it difficult to handle cluttered backgrounds and frequent target occlusion on underground belt conveyors, often resulting in bounding box offsets or low category confidence scores. (4) Insufficient bounding box regression accuracy during training: From the perspective of relative entropy (KL divergence), the original CIoU loss function lacks sufficient constraints for precise geometric center alignment, making it prone to offset errors, particularly in low-light or multi-scale target localization. These issues are not isolated. Illumination degradation exacerbates feature distortion in multi-scale targets, causing the weak features (low-entropy signals) of small-scale foreign objects to be overwhelmed by high-entropy noise in the feature pyramid. Feature fusion misalignment further amplifies the localization errors caused by low-light conditions, while imprecise regression loss accumulates these errors, ultimately compromising detection performance. Essentially, foreign object detection on belt conveyors is a process of extracting low-entropy target information from a high-entropy complex underground scene, achieving entropy reduction and enhanced safety order in the industrial system.

To address these issues, we improve the YOLOv11n baseline model and propose a complementary, collaboratively optimized model named SIRI-YOLO. Specifically, SIRI-YOLO introduces a self-calibrating illumination network (SCINet) to enhance the detail information of low-quality images, thereby improving the model’s adaptability to low-light environments. The original C2PSA module in YOLOv11n is upgraded to C2PSA-IRMB, which incorporates the inverted residual block attention mechanism (IRMB) to fuse local details with global dependencies, enhancing the model’s multi-scale target perception. The neck structure is reconstructed using an improved reparameterized generalized feature pyramid network (RepGFPN), which strengthens the fusion of high-level semantic information and low-level spatial features, leading to better target localization accuracy. Additionally, the Inner-MPDIoU (Inner Minimum Point Distance Intersection over Union) loss function is adopted to replace the original CIoU loss, further boosting localization performance. Notably, SIRI-YOLO achieves improved detection capability for low-quality images and multi-scale targets without significantly increasing the number of parameters, while still meeting real-time requirements.

## 2. YOLOv11n

YOLOv11 is a target detection framework in the YOLO series introduced in 2024. It inherits the decoupled head and Anchor-Free design concepts and incorporates multiple optimizations at the network structure level. YOLOv11n is the model with the fewest parameters and the lowest computational complexity, offering faster inference speed while maintaining high detection accuracy. Considering the real-time and lightweight requirements for foreign object detection on belt conveyors in underground coal mines, this paper selects YOLOv11n as the baseline model [12,13].

**Figure 1:**
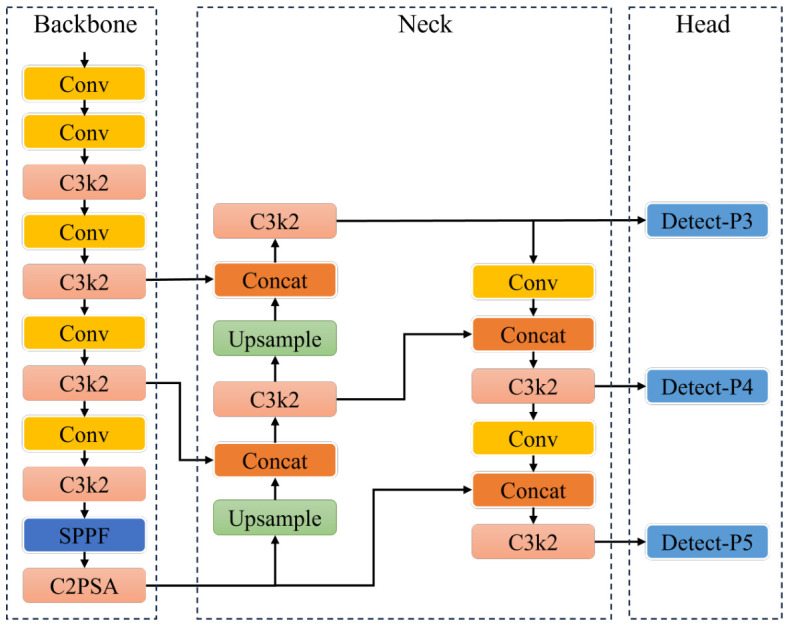
YOLOv11n Model Structure Diagram.

In the feature extraction stage, YOLOv11n retains the conventional convolution and SPPF modules while replacing the C2f module in YOLOv8 with the C3k2 module, which reduces computational load and enhances feature representation capability. Additionally, the C2PSA module is introduced into the backbone network to strengthen attention to key feature regions through a multi-head self-attention mechanism. In the feature fusion stage, YOLOv11n adopts the FPN+PAN structure and continues to use the C3k2 module for feature processing to achieve multi-scale feature fusion. In the detection head section, YOLOv11n employs a decoupled head and Anchor-Free design, optimizing classification and regression independently. The assignment of positive and negative samples follows a task-aligned allocation strategy. The classification branch uses binary cross-entropy loss, while the regression branch combines complete intersection over union (CIoU) loss with distribution focal loss, ensuring consistency between classification and regression tasks and thereby improving the accuracy of prediction results [14,15]. The network structure of YOLOv11n is illustrated in Figure 1.

## 3. SIRI-YOLO

**Figure 2:**
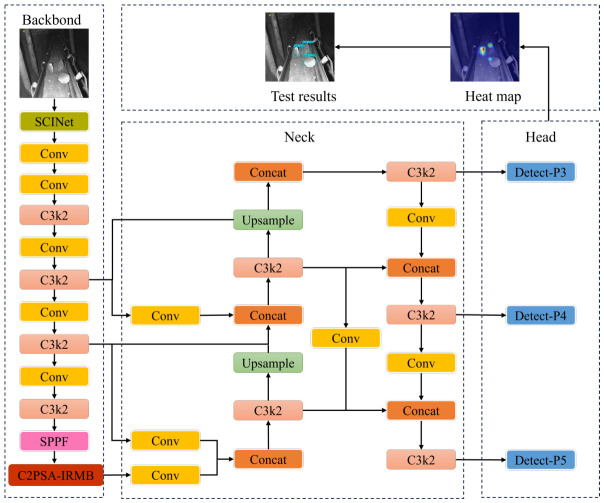
SIRI-YOLO Model Structure Diagram.

The structure diagram of the SIRI-YOLO model is shown in Figure 2. This model, built upon YOLOv11n, has been collaboratively improved to address the challenges encountered in foreign object detection on underground belt conveyors: (1) An SCINet module is introduced at the input stage. Through multi-stage progressive illumination optimization and a self-calibration mechanism, it restores the information entropy of low-quality images and reduces entropy loss of detail information, thereby enhancing the detection head’s response to foreign objects. (2) The C2PSA module is enhanced to C2PSA-IRMB. While retaining the dual-branch structure, the PSABlock is replaced with the IRMB module. By integrating local details with global dependencies, this design improves the utilization efficiency of multi-scale features and reduces ineffective entropy increase during feature extraction, without significantly increasing the number of parameters. (3) The neck structure is reconstructed using an improved RepGFPN, retaining the C3k2 module to control computational complexity. This strengthens the multi-scale interaction between high-level semantics and low-level spatial details, thereby reducing information entropy loss during feature pyramid transfer and mitigating the loss of effective information caused by feature fusion misalignment, while improving feature representation and target localization capabilities. (4) The InnerMPDIoU loss function is introduced to replace the original CIoU. From the perspective of relative entropy (KL divergence), it minimizes the distribution discrepancy between the predicted box and the ground-truth box. By precisely constraining the geometric center and corner positions, it achieves more accurate entropy minimization optimization, thereby enhancing the accuracy of bounding box regression.

### 3.1. SCINet Module

In coal mines, the underground environment is typically devoid of natural light, with illumination provided mainly by fixed or mobile artificial sources. As a result, the image data collected for foreign object detection on belt conveyors often suffer from issues such as uneven illumination distribution and low contrast, leading to degraded image quality and effective information entropy degradation. In such scenarios, key information within the captured images may not be effectively extracted due to the excessive proportion of high-entropy background noise, thereby affecting the target detection algorithm and preventing it from accurately identifying foreign objects being transported by the belt conveyor. To address these issues, this paper introduces the Self-Calibrating Illumination Network (SCINet) at the input stage to preprocess the original image data. Through a multi-stage self-calibration mechanism, the network assesses image quality and initiates the image enhancement operation within the SCINet module only when the overall grayscale mean of the image falls below a preset threshold; this process restores the information entropy of low-quality images and reduces the entropy loss of detail information. Normal images are processed neutrally to ensure that overall recognition accuracy remains unaffected. The introduction of the SCINet module provides more reliable, high-information-entropy input feature maps for the detection of foreign objects on belt conveyors.

**Figure 3:**
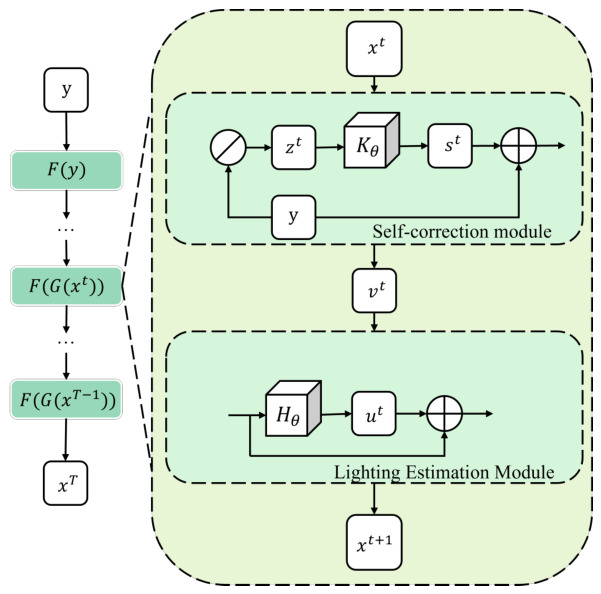
SCINet Module Structure Diagram.

The SCINet module can be divided into two parts: the illumination estimation module and the self-correction module. The structural diagram is shown in Figure 3. The lighting estimation module adopts a cascaded lighting learning strategy and introduces a weight sharing mechanism. It reuses the same network structure and parameters in multiple stages, thereby significantly reducing the model complexity and effectively enhancing the enhancement effect of images under low-lighting conditions [16]. The self-correction module is used to ensure that the outputs of each stage gradually converge, which can reduce the number of model parameters and enhance the stability of the network [17]. Through this self-calibrated illumination optimization, the SCINet module restores the information entropy of low-quality images and reduces the entropy loss of detail information. The SCINet module is mainly based on the Retinex theory. There is a connection between the low-light image y and the expected clear image z, as shown in Equation (1).

(1) y=z⊗x,

where *y* is the low-light image, *z* is the desired clear image, and *x* is the illumination component.

The lighting estimation module employs a cascaded structure for progressive lighting optimization. Through multiple processing stages with shared weights, it gradually improves image quality and optimizes global illumination, thereby progressively restoring the information entropy of the image. The lighting estimation process for each stage is given by Equation (2).

(2) $$F\left(x^{t}\right):\left\{\begin{array}{cc} u^{t}=H_{\theta }\left(x^{t}\right), & x^{0}=y\\ x^{t+1}=x^{t}+u^{t} & \end{array}\right.$$

where $F(x^{t})$ is the lighting estimation process in the *t*-th stage, $u^{t}$ is the residual in the *t*-th stage, $H_{\theta }$ is the mapping function with parameters θ, $x^{t}$ is the lighting component in the *t*-th stage, $x^{0}$ is the initial lighting component, and $x^{t+1}$ is the lighting component in the (*t* + 1)-th stage.

Through the progressive illumination optimization process described in Equation (2), image quality is further improved at each optimization stage, enabling accurate extraction of object features in dark areas while progressively restoring the information entropy of the image. However, the image enhancement process in Equation (2) may lead to the loss of image details and color distortion, i.e., an unintended entropy loss of fine-grained information. To address this issue, a self-correction module is introduced to correct the outputs at each stage, as shown in Equation (3), thereby mitigating such entropy loss and preserving effective information.

(3) $$G\left(x^{t}\right):\left\{\begin{array}{c} z^{t}=y⊙x^{t}\\ s^{t}=K_{\theta }\left(z^{t}\right)\\ v^{t}=y+s^{t} \end{array}\right.$$

where $z^{t}$ is the target image to be corrected in the *t*-th stage, $s^{t}$ is the image after self-correction mapping, $K_{\theta }$ is the parameter operator, and $v^{t}$ is the calibrated image used as input for the next stage.

The operating mode of the SCINet module is given by Equation (4). The illumination estimation module and the self-correction module work in tandem, where the input of each stage indirectly influences the output of that stage, thereby enabling convergence between stages. This allows the model to recover lost image details and restore the information entropy of low-quality images through image enhancement by the SCINet module under low-light conditions, reducing the entropy loss of detail information, improving image quality, and increasing the reliability of the model for foreign object detection on belt conveyors [18].

(4) $$F\left(x^{t}\right)\to F\left(G\left(x^{t}\right)\right)$$

**Figure 4:**
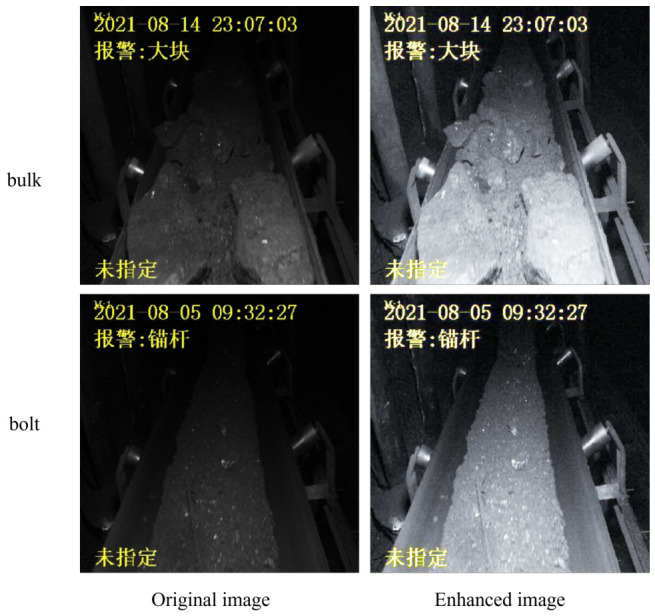
SCINet image enhancement effect diagram (The non-English terms in the figure are watermarks left during the data set collection process and have no impact on this article).

As shown in Figure 4, the comparison of the bulk and bolt before and after SCINet-based image enhancement reveals that the enhanced images exhibit richer details and more uniform brightness distribution. Compared to the original images, which suffer from uneven brightness, the textures in the enhanced images are clearer, allowing better observation of the shapes and finer details of both the bulk and the bolt. In the original images, the texture features at the edge of the bulk are difficult to distinguish from the normal coal flow on the conveyor belt under dim lighting conditions. In contrast, the enhanced images show a significantly improved distinction between the bulk edge and the surrounding coal flow. The bolt is often covered with coal powder and partially embedded in the coal flow, making it challenging to perform subsequent recognition and detection in low-light environments. After enhancement, however, the brightness of the bolt increases, greatly facilitating target detection. These enhancement results demonstrate that the addition of the self-calibrating illumination network (SCINet) can effectively improve the model’s detection accuracy in low-light environments.

### 3.2. IRMB Module

**Figure 5:**
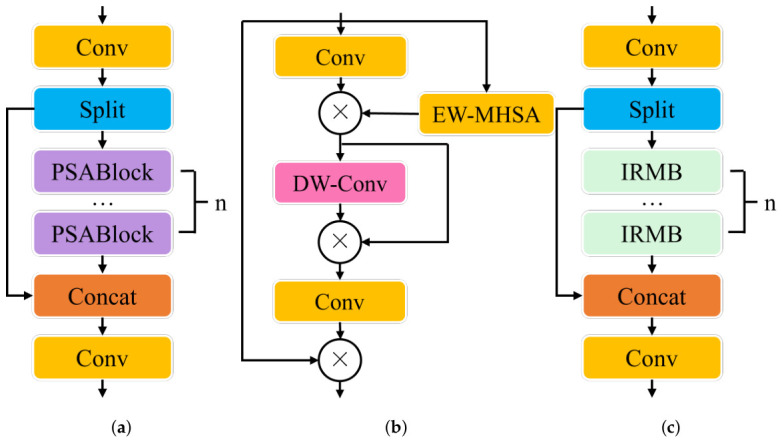
C2PSA-IRMB Module Structure Diagram. (**a**) C2PSA module. (**b**) IRMB module. (**c**) C2PSA-IRMB module.

The Inverted Residual Mobile Block (IRMB) combines a lightweight CNN structure with the dynamic processing capabilities of the Transformer model. Its structure is shown in Figure 5b. IRMB integrates depthwise separable convolution (3 × 3 DWConv) and multi-head self-attention (EW-MHSA). The 1 × 1 convolution is used for channel compression and expansion to optimize computational efficiency. The multi-head self-attention (EW-MHSA) preserves global context dependencies while reducing computational complexity. Depthwise separable convolution (DWConv) extracts local spatial detail features, thereby enhancing the model’s sensitivity to fine-grained features. By integrating local details with global dependencies, the IRMB module improves the utilization efficiency of multi-scale features and reduces ineffective entropy increase during feature extraction [19,20].

This paper integrates the IRMB module into the C2PSA module of YOLOv11n, thereby constructing an improved C2PSA-IRMB module, as shown in Figure 5c. The C2PSA-IRMB module retains the dual-branch structure of C2PSA and replaces the PSABlock module with the IRMB module. By stacking multiple IRMB modules, the model progressively refines complex semantics in the branches and finally merges them with the skip-connected branch, significantly enhancing the model’s perception of multi-scale targets while improving the utilization efficiency of multi-scale features and reducing ineffective entropy increase during feature extraction. This design avoids a substantial increase in the number of parameters while improving feature representation capability, making it particularly suitable for dense prediction tasks on embedded devices deployed for foreign object detection on underground belt conveyors. It maintains efficient detection performance in computationally limited environments and allows the model to capture and exploit long-range dependencies while remaining lightweight. As a result, the model can run efficiently on resource constrained devices while maintaining or even improving prediction accuracy.

### 3.3. Improve the RepGFPN Module

The neck of YOLOv11n adopts a multi-layer feature fusion structure based on FPN+PAN. It fuses feature maps of different resolutions through upsampling and concatenation and then uses the C3k2 module to extract features from the fused feature maps. This module employs smaller convolution kernels, which reduces computational load while effectively improving detection performance for multi-scale targets. However, this structure still follows the traditional layer-by-layer feature extraction approach along a fixed path. When processing detection targets in complex environments, it is prone to position deviations or incomplete semantic information, i.e., information entropy loss during feature pyramid transfer and loss of effective information caused by feature fusion misalignment [21].

To address this limitation, this paper introduces the Reparameterized Generalized Feature Pyramid Network (RepGFPN), an improved structure developed based on GFPN. It inherits the advantages of multi-scale feature interaction from GFPN and replaces traditional convolution with the CSPStage module, effectively enhancing the model’s representational capacity. Moreover, this structure adopts a training-inference decoupling design. During training, a multi-branch structure is used to strengthen the network’s feature learning ability, while during inference, the network is simplified to a single-branch convolution through reparameterization techniques, significantly improving inference speed [22]. By strengthening the fusion of high-level semantics and low-level spatial details, the improved RepGFPN reduces information entropy loss during feature pyramid transfer and mitigates the loss of effective information caused by feature fusion misalignment.

**Figure 6:**
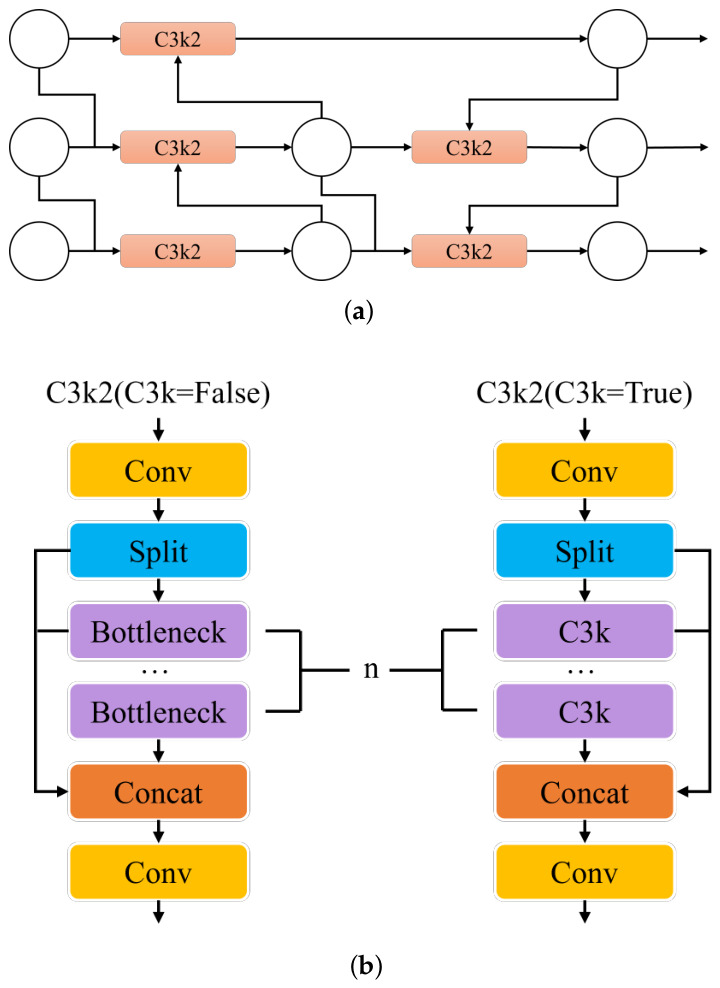
RepGFPN Module Structure Diagram. (**a**) RepGFPN module. (**b**) C3k2 module.

After improving RepGFPN, this paper adopts it as the Neck network of SIRI-YOLO. The structure of the improved RepGFPN is shown in Figure 6. Since the CSPStage module used in RepGFPN stacks multiple convolutional layers, it increases model complexity. In contrast, the C3k2 module in YOLOv11n concatenates multiple Bottleneck layers and uses smaller-kernel convolutions to optimize network parameters, thereby reducing computational complexity. Therefore, in the improved RepGFPN, we retain the overall structure of RepGFPN while still using the C3k2 module from YOLOv11n to replace the CSPStage module. The use of this improved RepGFPN enhances the network’s ability to capture high-level semantic features and low-level spatial details, thus further improving the model’s feature representation and target localization capabilities while reducing information entropy loss during feature pyramid transfer and mitigating the loss of effective information caused by feature fusion misalignment.

### 3.4. InnerMPDIoU Loss

During model training, the bounding box regression loss directly affects the positioning accuracy of the predicted boxes. In YOLOv11n, the default loss function is the CIoU loss function, as shown in Equation (5), which lacks a direct constraint from the perspective of relative entropy (KL divergence) to minimize the distribution discrepancy between the predicted box and the ground-truth box.

(5) $$L_{CIoU}=1-IoU+\frac{\rho^{2}\left(b,b^{gt}\right)}{c^{2}}+\alpha v$$

where IoU is the intersection-over-union ratio between the predicted box and the ground-truth box, ρ is the Euclidean distance between the center of the predicted box and the center of the ground-truth box, *c* is the diagonal length of the minimum enclosing rectangle of the predicted box and the ground-truth box, α is the trade-off parameter, and *v* is used to measure the consistency of the aspect ratio of the predicted box.

CIoU addresses the issue of gradient vanishing in traditional IoU loss for nonoverlapping bounding boxes by introducing center point distance and aspect ratio constraints. However, it remains difficult to handle foreign object detection on belt conveyors in the specific underground coal mine environment. From the perspective of relative entropy (KL divergence), CIoU does not directly minimize the distribution discrepancy between the predicted box and the ground-truth box, which can lead to gradient imbalance. On one hand, when the aspect ratio difference between the predicted box and the ground-truth box is large, the aspect ratio term may cause significant gradient fluctuations, thereby affecting the stability of model training. On the other hand, CIoU only constrains the Euclidean distance of the center point and does not account for precise corner position matching. Under low-light conditions or when target edges are blurred, even a small misalignment of the center point can be amplified, thus degrading localization accuracy [23].

In response to the limitations of CIoU, this paper introduces the InnerMPDIoU loss function [24]. From the perspective of relative entropy (KL divergence), this loss function minimizes the distribution discrepancy between the predicted box and the ground-truth box. It further enhances precise alignment of the bounding box’s geometric center based on MPDIoU, using the differences in the top-left and bottom-right coordinates between the ground-truth box and the predicted box as constraints. This makes the regression process focus more on the geometric shape of the bounding box, achieving more accurate entropy minimization optimization and alleviating the gradient imbalance problem of CIoU. The definition of the InnerMPDIoU loss function is shown in Equation (6).

(6) $$L_{\mathrm{InnerMPDIoU}}=1-IoU+\frac{d_{1}^{2}+d_{2}^{2}}{w^{2}+h^{2}}$$

where $d_{1}$ and $d_{2}$ are the Euclidean distances between the corresponding corner points of the ground-truth bounding box and the predicted bounding box, as defined in Equation (7). *w* and *h* are the width and height of the true bounding box, respectively.

(7) $$\left\{\begin{array}{cc} d_{1} & =\sqrt{{\left(x_{1}-x_{1}^{gt}\right)}^{2}+{\left(y_{1}-y_{1}^{gt}\right)}^{2}}\\ d_{2} & =\sqrt{{\left(x_{2}-x_{2}^{gt}\right)}^{2}+{\left(y_{2}-y_{2}^{gt}\right)}^{2}} \end{array}\right.$$

where $\left(x_{1},y_{1}\right)$ and $\left(x_{2},y_{2}\right)$ are the coordinates of the top-left and bottom-right corners of the predicted bounding box, while $\left(x_{1}^{gt},y_{1}^{gt}\right)$ and $\left(x_{2}^{gt},y_{2}^{gt}\right)$ are the corresponding corner point coordinates of the ground-truth bounding box.

Compared with CIoU, InnerMPDIoU explicitly constrains the positions of the four corner points of both the predicted and ground-truth bounding boxes, enabling the regression process to directly optimize the overall shape of the bounding box. From the perspective of relative entropy (KL divergence), this direct constraint on the joint distribution of corner points achieves more accurate entropy minimization, avoiding the gradient imbalance problem caused by the aspect ratio term in CIoU. The corner point constraints also restrict the center point position and the side lengths of the rectangle, achieving a more comprehensive geometric matching than using only the center point distance. Moreover, the InnerMPDIoU loss function calculates only the Euclidean distance between corner points and does not require additional inverse trigonometric functions, which to some extent improves inference speed.

## 4. Experimental Verification

### 4.1. Experimental Environment

The experimental software was developed using PyCharm (version 2022.2) as the integrated development environment. To ensure the reliability of the experimental results, all comparative and ablation experiments were conducted on a unified software and hardware platform. Specifically, the experiments were performed on a workstation equipped with an Intel Xeon Silver 4214R CPU, 32 GB of RAM, and a single NVIDIA RTX 3080Ti GPU with 12 GB of video memory. The operating system was Linux Ubuntu 20.04. The software environment consisted of CUDA 11.3, Python 3.8, and PyTorch 1.11.0. The complete experimental configuration is summarized in Table 1.

Based on the experimental environment and the performance of the equipment, the overall model training for this experiment selected the SGD optimizer with an initial learning rate of 0.01. The learning rate follows a linear decay schedule, decreasing from 0.01 to 0.0001 (i.e., lr0 × lrf, where lrf = 0.01) over the total 200 epochs. Cosine annealing is disabled. A warmup strategy is applied during the first 3 epochs, with warmup momentum set to 0.8 and warmup bias learning rate set to 0.1. The momentum and weight decay for the SGD optimizer are configured as 0.937 and 0.0005, respectively. The batch size is set to 32, and all input images are uniformly resized to 640 × 640 pixels. The data loading process uses 8 worker threads. An early stopping mechanism with a patience of 50 epochs is adopted to prevent overfitting. Additionally, label smoothing is disabled (0.0), and the loss function gains for box, class, and distribution focal loss (DFL) are set to 7.5, 0.5, and 1.5, respectively. Automatic mixed precision (AMP) is disabled to ensure training stability, and deterministic mode with a random seed of 0 is enabled for reproducibility. The detailed experimental parameters are shown in Table 2.

### 4.2. Data Set

The dataset used in this paper is derived from the coal-mine-specific video AI analysis dataset CUMT-BelT, provided by the Intelligent Detection and Pattern Recognition Research Center of China University of Mining and Technology, as well as from some publicly available datasets. A total of 2496 images containing large coal chunks and anchor rods were selected from these datasets and divided into a training set of 1998 images, a test set of 249 images, and a validation set of 249 images, following an 8:1:1 ratio.

To address the varying sizes of the original images due to different data sources, all images were resized to 640 × 640 during preprocessing. The LabelImg tool was then used to annotate the positions and categories of the targets, resulting in two classes: “bulk” and “bolt”. To prevent overfitting and improve model generalization, we applied several data augmentation strategies to the input images. In terms of color augmentation, random perturbations were introduced to the hue, saturation, and brightness. For geometric augmentation, random translations, scaling, and horizontal flips were performed. Additionally, mosaic augmentation was applied with a high probability, combining multiple images into one for training.

### 4.3. Evaluation Indicators

This paper uses the following evaluation metrics to assess model performance: average precision (mAP@50 and mAP@50:95), precision (P), recall (R), number of parameters, and frame rate (FPS).

(1) Mean Average Precision (mAP): The average of detection accuracy across all categories, derived from precision (P) and recall (R), as shown in Equation (8) [25].

(8) $$mAP=\frac{1}{n}\sum_{i=1}^{n}AP_{i}$$

where *AP* is the average detection accuracy for each category.

(2) Precision (P): The proportion of true positive samples among all samples predicted as positive by the model, as shown in Equation (9) [26].

(9) $$P=\frac{TP}{TP+FP}$$

where *TP* is the number of true positives (positive samples correctly predicted), and *FP* is the number of false positives (negative samples incorrectly predicted as positive).

(3) Recall (R): The proportion of true positive samples correctly detected out of all actual positive samples, as shown in Equation (10) [27].

(10) $$R=\frac{TP}{TP+FN}$$

where *FN* is the number of false negatives (positive samples incorrectly predicted as negative).

(4) Frame Rate (FPS): The number of image frames refreshed per second, as shown in Equation (11) [28].

(11) $$FPS=\frac{FrameNum}{ElapsedTime}$$

where *FrameNum* is the total number of images, and *ElapsedTime* is the time taken to detect all these images.

### 4.4. Performance Comparison

**Figure 7:**
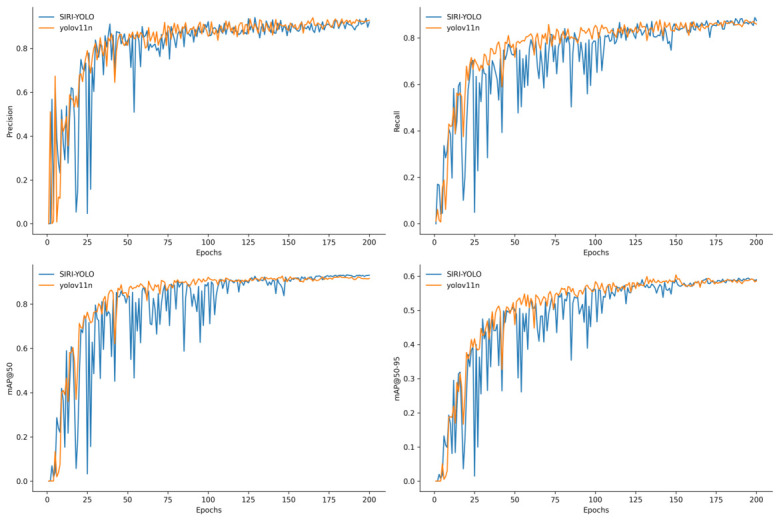
Performance comparison chart of SIRI-YOLO and YOLOv11n models.

Experiments were conducted on the dataset used in this paper to compare SIRI-YOLO with the baseline model YOLOv11n. The comparison results are shown in Figure 7. It can be observed that the mAP@50, mAP@50:95, precision, and recall of SIRI-YOLO all outperform those of the baseline model.

Compared with the original YOLOv11n, SIRI-YOLO exhibits more pronounced fluctuations during training. The primary reason is that the improvements introduced in this work do not simply reduce model complexity; instead, they incorporate multiple “enhancement–fusion–constraint” modules, which increase the complexity of feature learning and gradient optimization.

First, SCINet employs a multi-stage progressive illumination optimization and self-correction mechanism that effectively enhances low-light image quality. However, this gradual enhancement alters the input feature distribution, potentially causing noticeable output fluctuations in the early training stage. Moreover, image enhancement itself may lead to detail loss and color deviation, forcing the network to converge under more intricate input conditions.

Second, IRMB combines depthwise separable convolution with multi-head self-attention. While this improves both local detail extraction and global dependency modeling, it also makes feature representation and gradient propagation paths more complex, thereby increasing the training process’s sensitivity to data variations.

Third, RepGFPN adopts a multi-branch feature interaction structure during training, which strengthens the fusion of high-level semantics and low-level details. However, multi-branch learning typically introduces more distinct periodic fluctuations in the loss curve.

Finally, InnerMPDIoU applies stricter geometric constraints to the four corner points of the bounding box compared to CIoU, achieving more precise regression. When the target aspect ratio differs significantly or the boundary is ambiguous, the gradient changes become more sensitive, resulting in more obvious oscillations during training.

Given that our dataset suffers from issues such as low illumination, strong background noise, and multi-scale targets, such fluctuations are understandable. Importantly, the model still converges stably and achieves better overall performance, indicating that the observed fluctuations primarily stem from stronger feature reconstruction and stricter optimization constraints, rather than from unstable training.

### 4.5. Comparative Experiment

To verify whether the SIRI-YOLO model improves the detection performance of foreign objects on belt conveyors, a comparative experiment was conducted using the same experimental environment, parameters, and dataset with the widely used object detection algorithms YOLOv3-tiny, YOLOv5n, YOLOv6n, YOLOv8n, YOLOv9s, YOLOv10n, YOLOv11n, RT-DETR-r18, Faster-RCNN, SSD and SDGW-YOL0v11. The experimental results are shown in Table 3.

As shown in Table 3, the SIRI-YOLO model proposed in this paper achieves the highest values across all four evaluation metrics: mAP@50, mAP@50:95, precision, and recall reach 92.8%, 59.4%, 89.5%, and 87.2%, respectively. In terms of the number of parameters, SIRI-YOLO has 2.92 M, which is slightly higher than those of YOLOv5n, YOLOv10n, and YOLOv11n, but its detection accuracy is significantly better than that of these lightweight models. Compared with YOLOv5n, SIRI-YOLO shows improvements of 3.3%, 2.3%, and 3.0% in mAP@50, mAP@50:95, and recall, respectively, with an increase of only 0.41 M in parameters. Compared with YOLOv11n, the corresponding improvements are 3.3%, 1.1%, and 1.6%, with a parameter increase of 0.34 M. These results demonstrate a favorable balance between detection accuracy and model lightweighting.

Compared with YOLOv8n and YOLOv9s, SIRI-YOLO also demonstrates clear advantages. Although YOLOv8n has a slightly lower parameter count, its mAP@50, precision, and recall are 4.1%, 0.7%, and 6.4% lower than those of SIRI-YOLO, respectively. YOLOv9s has a parameter count of 7.17 M, approximately 2.5 times that of SIRI-YOLO, yet still lags behind by 4.0%, 2.0%, and 2.9% in mAP@50, precision, and recall, respectively. In terms of detection speed, SIRI-YOLO achieves 70.01 f/s, which is slightly lower than that of extremely lightweight models such as YOLOv3-tiny and YOLOv5n but still satisfies the real-time detection requirements of underground coal mines.

When compared with non-YOLO models, the transformer-based RT-DETR-r18 can achieve relatively high frame rates under certain configurations. However, it has a complex structure with approximately 19.87 million parameters, and its mAP@50 is only about 87.2%, which is roughly 5.6 percentage points lower than that of SIRI-YOLO. The classic two-stage detector Faster-RCNN is sensitive to multi-scale targets and achieves a recall of 90.9%, but its precision is only 55.0%, indicating many false positives. Moreover, its parameter count reaches 137.10 million, and its inference speed is only 27.29 frames per second, making it difficult to meet the real-time detection requirements of underground environments. The traditional single-stage detector SSD employs a multi-scale feature design suitable for lightweight applications, yet its detection performance in complex low-light and multi-scale coal mine scenarios remains inferior to that of SIRI-YOLO, particularly in metrics such as mAP@50:95. Overall, while RT-DETR, Faster-RCNN, and SSD have unique advantages in other application domains, their comprehensive performance in this scenario falls short of SIRI-YOLO.

SDGW-YOLOv11 is an improved version of YOLOv11 specifically designed for coal conveyor belt scenarios. However, experimental results show that its mAP@50 is only 82.8%, substantially lower than the 92.8% achieved by SIRI-YOLO. This indicates that SIRI-YOLO effectively addresses challenges such as target occlusion, multi-scale variation, and feature alignment mismatch through modules like RepGFPN and InnerMPDIoU, thereby enhancing detection capabilities for weakly illuminated and multi-sized foreign objects. In contrast, SDGW-YOLOv11 offers limited accuracy improvement and cannot compete with the overall performance of SIRI-YOLO.

Overall, SIRI-YOLO significantly improves detection accuracy while maintaining a low parameter count and high detection speed. In particular, its outstanding recall performance indicates a lower missed detection rate for multi-scale foreign object targets, making it well-suited for practical applications of foreign object detection on underground belt conveyors.

**Figure 8:**
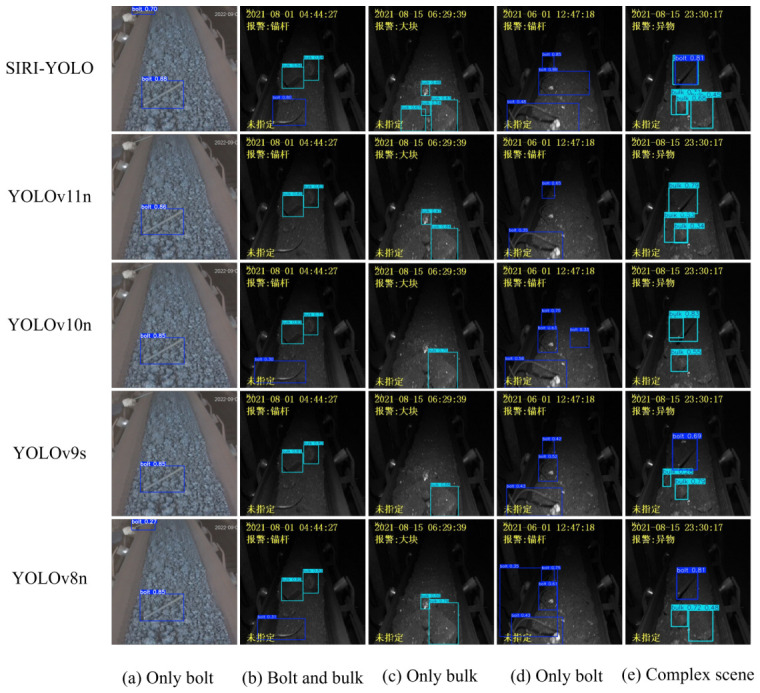
Different models’ detection results for foreign objects on belt conveyors (The non-English terms in the figure are watermarks left during the data set collection process and have no impact on this article).

To visually compare the detection performance of different models for foreign objects on belt conveyors, images containing “bulk” and “bolt” were selected from the test set and detected by different models. The results are shown in Figure 8.

The detection results show that, compared with the SIRI-YOLO model, YOLOv11n, YOLOv10n, YOLOv9s, and YOLOv8n all exhibit missed detections in poorly lit scenes, where the effective information entropy of targets is degraded and low-entropy target signals are overwhelmed by high-entropy background noise, with this phenomenon being most evident in Figure 8c. In Figure 8d, the YOLOv8n model produces a false detection, mistakenly identifying the edge of the belt conveyor as an anchor bolt, which is attributed to blurred features in distant and edge-occluded areas, i.e., information entropy loss caused by feature fusion misalignment. By comparison, the SIRI-YOLO model demonstrates superior accuracy in low-light and multi-scale target detection scenarios, effectively extracting low-entropy target information from high-entropy complex scenes. In the detection of large coal chunks shown in Figure 8b, SIRI-YOLO achieves a detection accuracy of 84%, which is 1%, 7%, 2%, and 2% higher than that of YOLOv11n, YOLOv10n, YOLOv9s, and YOLOv8n, respectively.

**Figure 9:**
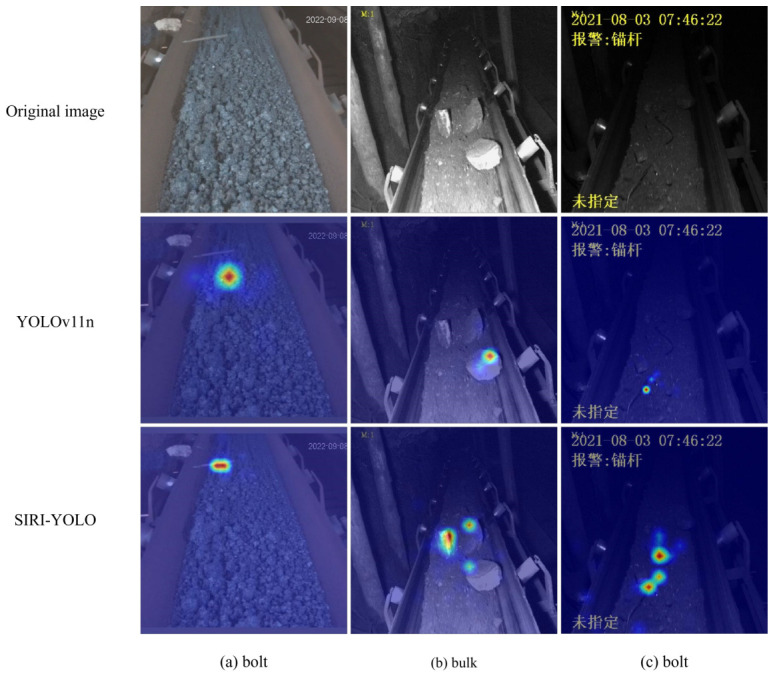
Comparison of visualized heat maps between the SIRI-YOLO model and the YOLOv11n model (The non-English terms in the figure are watermarks left during the data set collection process and have no impact on this article).

To visually evaluate the perception of the SIRI-YOLO model for key regions of foreign objects on the belt conveyor, a visualization experiment was conducted using EigenGradCAM. The results are shown in Figure 9.

In the generated heatmap, the darker red regions indicate areas that contribute most to the final detection result, where the model pays the most attention. In contrast, the blue regions represent features that have a smaller impact on target detection and are typically considered redundant information, i.e., high-entropy background noise or features with low contribution to target detection. The experimental results show that the improved YOLOv11n model exhibits scattered attention and insufficient focus on foreign objects such as large coal chunks and anchor rods, with instances of incorrectly focusing on non-target areas, indicating its difficulty in extracting low-entropy target information from high-entropy complex backgrounds. In comparison, the SIRI-YOLO model demonstrates significantly enhanced focus on foreign objects, with the ability to concentrate on more detailed target regions while generating less redundant information—i.e., effectively extracting low-entropy target information from high-entropy scenes and achieving system entropy reduction—and achieving better localization. Therefore, the proposed SIRI-YOLO model shows clear advantages in detecting multi-scale foreign objects on belt conveyors under low-light and complex conditions in underground coal mines, contributing to the safety entropy reduction of the industrial system.

To further evaluate the robustness and generalization capability of the proposed SIRI-YOLO model on other low-light and complex scene datasets, experiments were conducted on the publicly available ExDark dataset. ExDark is specifically designed for low-light object detection, comprising 12 categories with significant scale variations among the target instances and complex backgrounds. The dataset contains a total of 7363 images. To maintain consistency with the experimental setup described earlier, the dataset was re-partitioned into training, test, and validation sets with a ratio of 8:1:1, resulting in 5 891, 936, and 736 images, respectively. Under identical experimental parameters, SIRI-YOLO was compared against YOLOv11n, and the results are presented in Table 4.

SIRI-YOLO consistently outperforms the baseline YOLOv11n model on the ExDark dataset, achieving improvements of 4.2% in mAP@50, 2.8% in mAP@50:95, and 2.7% in recall. Although the absolute performance on ExDark is lower than that on the CUMT-BelT dataset due to differences in category diversity and scene complexity, the relative gains over the baseline remain significant. These results demonstrate that the proposed modules—SCINet for illumination enhancement, IRMB for multi-scale feature utilization, RepGFPN for feature fusion alignment, and InnerMPDIoU for precise bounding box regression—are not overfitted to a single dataset but instead provide robust improvements across different low-light and complex scenarios. Therefore, SIRI-YOLO possesses strong generalization capabilities and can effectively address multi-scale target detection issues in various low-light and complex scenarios.

### 4.6. Ablation Experiment

To verify the effectiveness of each individual improvement module and their collaborative effects, this paper progressively integrates SCINet, IRMB, RepGFPN, and InnerMPDIoU into YOLOv11n for ablation experiments. The results are presented in Table 5. Overall, each module does not simply enhance performance in isolation; rather, each addresses a specific type of “entropy loss” issue, including degradation under low-light input, insufficient multi-scale feature perception, mismatch in feature pyramid fusion, and inaccurate bounding box regression. Together, they form a progressive, collaborative optimization chain that spans input enhancement, feature extraction, feature fusion, and localization constraints.

The single-module experiments show that SCINet increases mAP@50 to 90.0%, indicating its ability to recover effective information entropy from low-quality images through self-calibrated illumination optimization. However, its mAP@50:95 and recall exhibit slight fluctuations, suggesting that enhancing only the input may alter the feature distribution. IRMB improves precision to 90.5% after introduction, demonstrating its advantage in integrating local details with global dependencies and enhancing multi-scale target perception. RepGFPN alone raises mAP@50 to 91.2% and precision to 89.6%, indicating a stronger compensatory effect on feature alignment and semantic interaction in complex scenes. InnerMPDIoU alone achieves an mAP@50 of 91.3% and recall of 86.0%, showing that stricter geometric constraints help improve bounding box regression accuracy and reduce localization errors.

Furthermore, the combination experiments reveal that the improved modules do not simply add up but instead exhibit significant complementary and synergistic relationships. Among the two-module combinations, SCINet+IRMB increases mAP@50 to 90.4% but reduces FPS to 51.56, indicating that while input enhancement and multi-scale feature modeling complement each other to improve detection accuracy, their combination also substantially increases the cost of feature extraction and gradient optimization. SCINet+RepGFPN and IRMB+RepGFPN achieve mAP@50 of 91.1% and 91.3%, respectively. Among these, IRMB+RepGFPN delivers better precision, mAP@50:95, and overall performance. This shows that RepGFPN amplifies the effect of multi-scale perception modules in alleviating feature fusion mismatches and enhancing semantic and spatial information interaction. Notably, RepGFPN+InnerMPDIoU achieves the best results among all two-module combinations, with mAP@50, mAP@50:95, precision, and recall reaching 91.6%, 59.9%, 89.0%, and 85.9%, respectively. This indicates a direct synergy between high-quality feature fusion and precise boundary constraints.

Specifically, when SCINet, IRMB, and RepGFPN are combined, the model’s mAP@50 increases to 91.9%, demonstrating that SCINet first restores information entropy in low-light images, IRMB then models multi-scale semantics, and RepGFPN finally reduces information entropy loss during feature pyramid transfer. Together, they form a collaborative pipeline of “input restoration → feature enhancement → fusion correction”. On this basis, further introducing InnerMPDIoU yields the best overall performance, with mAP@50, mAP@50:95, precision, and recall reaching 92.8%, 59.4%, 89.5%, and 87.2%, respectively. The parameter count is only 2.92M, and FPS remains at 70.01, indicating that more precise bounding box regression can amplify the benefits of previous modules in feature representation and localization without compromising real-time performance. It is worth noting that while some module combinations further enhance feature reconstruction and constraint capabilities, they also introduce some inference overhead. This suggests that the proposed method maintains a favorable balance between accuracy improvement and computational cost, meeting the dual requirements of lightweight design and real-time performance in underground coal mine scenarios.

## 5. Conclusions

This paper addresses the challenges in foreign object detection on belt conveyors, including poor detection performance under low-light conditions caused by effective information entropy degradation and high-entropy background noise, difficulty in extracting multi-scale target features due to uneven information entropy distribution across scales, and insufficient detection accuracy in complex environments. Based on YOLOv11n, an improved model named SIRI-YOLO is proposed. The main conclusions are as follows:

(1) The model enhances detail information and restores information entropy in low-quality images through the SCINet module, integrates local and global features via the C2PSA-IRMB module to improve multi-scale perception while reducing ineffective entropy increase, strengthens feature fusion using the improved RepGFPN to reduce information entropy loss during feature pyramid transfer, and introduces the InnerMPDIoU loss function to optimize bounding box regression accuracy from the perspective of relative entropy (KL divergence) for more accurate entropy minimization. Experimental results show that SIRI-YOLO achieves an mAP@50 of 92.8%, an mAP@50:95 of 59.4%, a precision of 89.5%, and a recall of 87.2%, representing improvements of 3.3%, 1.1%, 5.6%, and 1.6%, respectively, over the baseline YOLOv11n model. The model size is only 2.92 M parameters, and the frame rate remains at 70.01 fps, striking a good balance between accuracy and lightweight design.
(2) Compared with mainstream models such as YOLOv3-tiny, YOLOv5n, YOLOv6n, YOLOv8n, YOLOv9s, YOLOv10n, YOLOv11n, RT-DETR-r18, Faster-RCNN, SSD and SDGW-YOL0v11, SIRI-YOLO achieves the best performance in terms of mAP@50, mAP@50:95, precision, and recall, demonstrating its superiority in extracting low-entropy target information from high-entropy complex scenes. While the number of parameters increases slightly, the detection speed remains at an average level, demonstrating excellent overall performance. On the public ExDark low-light dataset, SIRI-YOLO improves mAP@50 by 4.2% over YOLOv11n, demonstrating strong generalization across different low-light and complex scenarios.
(3) With a smaller model size and higher detection accuracy, this model effectively addresses the challenges of uneven illumination, significant variations in target scale, and complex backgrounds in foreign object detection. By achieving system entropy reduction through accurate foreign object detection, it enhances the safety and order of the coal mine industrial system. The model is well suited for deployment on edge computing platforms in underground coal mines and satisfies the dual requirements of real-time performance and reliability for belt conveyor foreign object detection.

## Figures and Tables

**Table 1:** Experimental environment.

Configuration	Parameters
CPU	Intel Xeon Silver 4214R
GPU	NVIDIA RTX 3080Ti (12G)
RAM	32G
Operating System	Linux Ubuntu 20.04
CUDA	CUDA 11.3
Python	Python 3.8
PyTorch	PyTorch 1.11.0
IDE	CUDA PyCharm 2022.2

**Table 2:** Experimental parameters.

Parameter	Value
epochs	200
optimize	SGD
lr0	0.01
lrf	0.01
batch size	32
momentum	0.937
workers	8
input image size	640 × 640
patience	50

**Table 3:** Comparison results of different models.

Model	mAP@50	mAP@50:95	P	R	Params/M	FPS/f·s^−1^
YOLOv3-tiny [29]	87.2	55.2	82.9	84.4	12.13	174.85
YOLOv5n [30]	89.5	57.1	89.0	84.2	2.51	92.62
YOLOv6n [31]	90.1	58.4	88.3	82.0	4.23	112.11
YOLOv8n [32]	88.7	56.9	88.8	80.8	3.01	101.34
YOLOv9s [33]	88.8	59.3	87.5	84.3	7.17	48.14
YOLOv10n [34]	85.9	54.8	82.5	81.4	2.27	90.86
YOLOv11n [35]	89.5	58.3	83.9	85.6	2.58	77.09
RT-DETR-r18 [36]	87.2	55.8	86.1	82.6	19.87	119.22
Faster-RCNN [37]	88.2	45.5	55.0	90.9	137.10	27.29
SSD [38]	86.1	48.3	85.3	81.3	26.29	19.27
SDGW-YOL0v11 [39]	82.8	40.3	78.9	83.7	2.74	42.71
SIRI-YOLO	92.8	59.4	89.5	87.2	2.92	70.01

**Table 4:** The experimental comparison results of SIRI-YOLO and the base model on the CUMT-BelT dataset and the ExDark dataset.

Model	Dataset	mAP@50	mAP@50:90	P	R
YOLOv11n	CUMT-BelT	89.5	58.3	83.9	85.6
SIRI-YOLO		92.8	59.4	89.5	87.2
YOLOv11n	ExDark	54.6	32.2	63.3	51.9
SIRI-YOLO		58.8	35.0	62.4	54.6

**Table 5:** The results of the ablation experiment.

YOLOv11n	SCINet	IRMB	RepGFPN	InnerMPDIoU	mAP@50	mAP@50:95	P	R	Params/M	FPS/f·s^−1^
✓					89.5	58.3	83.9	85.6	2.58	77.09
✓	✓				90.0	55.7	87.8	82.4	2.58	78.24
✓		✓			91.1	59.0	90.5	84.3	2.60	80.13
✓			✓		91.2	58.9	89.6	83.4	2.89	72.45
✓				✓	91.3	58.7	83.5	86.0	2.58	79.02
✓	✓	✓			90.4	58.9	88.5	84.0	2.60	51.56
✓	✓		✓		91.1	57.8	88.0	85.4	2.91	71.09
✓		✓	✓		91.3	58.4	88.8	84.0	2.91	64.12
✓			✓	✓	91.6	59.9	89.0	85.9	2.89	74.99
✓	✓	✓	✓		91.9	59.0	88.1	82.5	2.92	66.34
✓		✓	✓	✓	92.2	58.1	86.9	85.2	2.91	65.09
✓	✓	✓	✓	✓	92.8	59.4	89.5	87.2	2.92	70.01

## Data Availability

The original contributions presented in this study are included in the article. Further inquiries can be directed to the corresponding author.

## References

[1] Wang Z., Liu Y., Sun X., Ding X., Zhao H., Li L., Song B., Huang J., Li G., Zhou Y. (2026). Progress and prospect of intelligent mining technology and equipment for open-pit coal mines. Coal Sci. Technol..

[2] Dai Y., Liu W. (2023). GL-YOLO-Lite: A novel lightweight fallen person detection model. Entropy.

[3] Zhou Z., Wang S., Wang X., Zheng W., Xu Y. (2025). YOLO-GRBI: An Enhanced Lightweight Detector for Non-Cooperative Spatial Target in Complex Orbital Environments. Entropy.

[4] Li S., Liu Y., Li M., Ding L. (2023). DF-YOLO: Highly accurate transmission line foreign object detection algorithm. IEEE Access.

[5] Singh S., Kumari R., Pallavi P., Saurabh P. (2026). A systematic review of deep learning methods for low-light image enhancement and object detection. Discov. Appl. Sci..

[6] Yin X., Yu Z., Fei Z., Lv W., Gao X., Iliadis L., Papaleonidas A., Angelov P., Jayne C. (2023). PE-YOLO: Pyramid Enhancement Network for Dark Object Detection. Proceedings of the Artificial Neural Networks and Machine Learning—ICANN 2023; Lecture Notes in Computer Science.

[7] Hashmi K.A., Kallempudi G., Stricker D., Afzal M.Z. (2023). FeatEnHancer: Enhancing Hierarchical Features for Object Detection and Beyond Under Low-Light Vision. Proceedings of the IEEE/CVF International Conference on Computer Vision (ICCV).

[8] Shi L., Yang C., Liu X., Zhou X. (2025). Lightweight low-light object detection algorithm based on CDD-YOLO. Comput. Eng. Appl..

[9] Lin T., Huang G., Yuan X., Zhong G., Huang X., Pun C. (2024). SCDet: Decoupling discriminative representation for dark object detection via supervised contrastive learning. Vis. Comput..

[10] Schutera M., Hussein M., Abhau J., Mikut R., Reischl M. (2020). Night-to-day: Online image-to-image translation for object detection within autonomous driving by night. IEEE Trans. Intell. Veh..

[11] Gao L., Cao H., Zou H., Wu H. (2025). Dmn-yolo: A robust yolov11 model for detecting apple leaf diseases in complex field conditions. Agriculture.

[12] Zhou K., Jiang S. (2025). Forest fire detection algorithm based on improved YOLOv11n. Sensors.

[13] Zhang Y., Liu Z., Guo X., Li C., Teng G. (2025). Wheat Head Detection in Field Environments Based on an Improved YOLOv11 Model. Agriculture.

[14] Liu L., Sun T., Guo X., Yuan Z. (2026). Multi-Objective Detection of River and Lake Spaces Based on YOLOv11n. Sensors.

[15] Yang L., Cui W., Li J., Han G., Zhou Q., Lan Y., Zhao J., Qiao Y. (2025). A Real-Time Cotton Boll Disease Detection Model Based on Enhanced YOLOv11n. Appl. Sci..

[16] Yang J., Yue Z., Hu Y., Wang M., Zheng B. (2024). Thermal Defect Detection in Power Equipment Based on YOLOv8s-SCINet-GAM Model with Low-Light Image Enhancement Algorithm. Proceedings of the 2024 4th International Conference on Energy, Power and Electrical Engineering (EPEE), Wuhan, China, September 2024.

[17] Liu Y., Li S., Zhou L., Liu H., Li Z. (2025). Dark-Yolo: A low-light object detection algorithm integrating multiple attention mechanisms. Appl. Sci..

[18] Liu Y., Lu Y., Zhao X., Cui H., Zhang C., Fan H., Sun D., Ma H., Huang D., Chen W., Pan Y., Chen H. (2025). A New Target Detection Model in Complex Environments Based on Unmanned Aerial Vehicle Images. Proceedings of the Advanced Intelligent Computing Technology and Applications; Lecture Notes in Computer Science.

[19] Yu Z. (2024). RT-DETR-iRMB: A Lightweight Real-Time Small Object Detection Method. Proceedings of the 2024 IEEE 6th Advanced Information Management, Communicates, Electronic and Automation Control Conference (IMCEC), Chongqing, China, May 2024.

[20] Zhu J., Wang T., Wang L., Luo Z., Zhang H., Li X., Hao T., Meng W., Wu Z., He Q. (2024). Transmission Line Equipment Defect Detection Based on Improved YOLO Network. Proceedings of the Neural Computing for Advanced Applications—5th International Conference, NCAA 2024, Guilin, China, 5–7 July 2024, Proceedings, Part III.

[21] Liu H., Li L., Li Y., Long Q., Chen Z. (2024). A Hybrid Attention Mechanism and RepGFPN Method for Detecting Wall Cracks in High-Altitude Cleaning Robots. IEEE Photonics J..

[22] Mei M., Zhou Z., Liu W., Ye Z. (2024). GOI-YOLOv8 grouping offset and isolated GiraffeDet low-light target detection. Sensors.

[23] Zhu G., Qi H., Lv K. (2025). Dgyolov8: An enhanced model for steel surface defect detection based on yolov8. Mathematics.

[24] Ding Y., Jiang C., Song L., Liu F., Tao Y. (2024). RVDR-YOLOv8: A weed target detection model based on improved YOLOv8. Electronics.

[25] Carrasco D.P., Rashwan H.A., Garcia M.A., Puig D. (2021). T-YOLO: Tiny vehicle detection based on YOLO and multi-scale convolutional neural networks. IEEE Access.

[26] Zhou J., Zhang B., Yuan X., Lian C., Ji L., Zhang Q., Yue J. (2023). YOLO-CIR: The network based on YOLO and ConvNeXt for infrared object detection. Infrared Phys. Technol..

[27] Vijayakumar A., Vairavasundaram S. (2024). Yolo-based object detection models: A review and its applications. Multimed. Tools Appl..

[28] Wang C., Wang Q., Qian Y., Hu Y., Xue Y., Wang H. (2023). Dp-yolo: Effective improvement based on yolo detector. Appl. Sci..

[29] Yu Z., Bouganis C.S., Rincon F., Barba J., So H.K.H., Diniz P., Caba J. (2020). A Parameterisable FPGA-Tailored Architecture for YOLOv3-Tiny. Proceedings of the Applied Reconfigurable Computing. Architectures, Tools, and Applications; Lecture Notes in Computer Science.

[30] Bie M., Liu Y., Li G., Hong J., Li J. (2023). Real-time vehicle detection algorithm based on a lightweight You-Only-Look-Once (YOLOv5n-L) approach. Expert Syst. Appl..

[31] Wang H., Xu X., Liu Y., Lu D., Liang B., Tang Y. (2023). Real-time defect detection for metal components: A fusion of enhanced Canny–Devernay and YOLOv6 algorithms. Appl. Sci..

[32] Wang Z., Hua Z., Wen Y., Zhang S., Xu X., Song H. (2024). E-YOLO: Recognition of estrus cow based on improved YOLOv8n model. Expert Syst. Appl..

[33] Shi Y., Duan Z., Qing S., Zhao L., Wang F., Yuwen X. (2024). YOLOV9S-Pear: A lightweight YOLOV9S-Based improved model for young Red Pear Small-Target recognition. Agronomy.

[34] Huang Y., Liu Z., Zhao H., Tang C., Liu B., Li Z., Wan F., Qian W., Qiao X. (2025). YOLO-YSTs: An improved YOLOv10n-based method for real-time field pest detection. Agronomy.

[35] Luo R., Zhao R., Ding X., Peng S., Cai F. (2025). High-precision complex orchard passion fruit detection using the phd-yolo model improved from yolov11n. Horticulturae.

[36] Jun E.L.T., Tham M.L., Kwan B.H. (2024). A Comparative Analysis of RT-DETR and YOLOv8 for Urban Zone Aerial Object Detection. Proceedings of the 2024 IEEE International Conference on Automatic Control and Intelligent Systems (I2CACIS), Shah Alam, Malaysia, June 2024.

[37] Guan B., Wu Y., Zhu J., Kong J., Dong W. (2025). GC-Faster RCNN: The object detection algorithm for agricultural pests based on improved hybrid attention mechanism. Plants.

[38] Tan L., Wu H., Xu Z., Xia J. (2025). Multi-object garbage image detection algorithm based on SP-SSD. Expert Syst. Appl..

[39] Shaokai Y., Lihong D., Yi Q. (2025). Foreign object detection for underground coal mine conveyor belts in occlusion scenarios based on SDGW-YOLOv11. Electr. Meas. Technol..

